# Predicted Influences of Artificial Intelligence on Nursing Education: Scoping Review

**DOI:** 10.2196/23933

**Published:** 2021-01-28

**Authors:** Christine Buchanan, M Lyndsay Howitt, Rita Wilson, Richard G Booth, Tracie Risling, Megan Bamford

**Affiliations:** 1 Registered Nurses' Association of Ontario Toronto, ON Canada; 2 Arthur Labatt Family School of Nursing Western University London, ON Canada; 3 College of Nursing University of Saskatchewan Saskatoon, SK Canada

**Keywords:** nursing, artificial intelligence, education, review

## Abstract

**Background:**

It is predicted that artificial intelligence (AI) will transform nursing across all domains of nursing practice, including administration, clinical care, education, policy, and research. Increasingly, researchers are exploring the potential influences of AI health technologies (AIHTs) on nursing in general and on nursing education more specifically. However, little emphasis has been placed on synthesizing this body of literature.

**Objective:**

A scoping review was conducted to summarize the current and predicted influences of AIHTs on nursing education over the next 10 years and beyond.

**Methods:**

This scoping review followed a previously published protocol from April 2020. Using an established scoping review methodology, the databases of MEDLINE, Cumulative Index to Nursing and Allied Health Literature, Embase, PsycINFO, Cochrane Database of Systematic Reviews, Cochrane Central, Education Resources Information Centre, Scopus, Web of Science, and Proquest were searched. In addition to the use of these electronic databases, a targeted website search was performed to access relevant grey literature. Abstracts and full-text studies were independently screened by two reviewers using prespecified inclusion and exclusion criteria. Included literature focused on nursing education and digital health technologies that incorporate AI. Data were charted using a structured form and narratively summarized into categories.

**Results:**

A total of 27 articles were identified (20 expository papers, six studies with quantitative or prototyping methods, and one qualitative study). The population included nurses, nurse educators, and nursing students at the entry-to-practice, undergraduate, graduate, and doctoral levels. A variety of AIHTs were discussed, including virtual avatar apps, smart homes, predictive analytics, virtual or augmented reality, and robots. The two key categories derived from the literature were (1) influences of AI on nursing education in academic institutions and (2) influences of AI on nursing education in clinical practice.

**Conclusions:**

Curricular reform is urgently needed within nursing education programs in academic institutions and clinical practice settings to prepare nurses and nursing students to practice safely and efficiently in the age of AI. Additionally, nurse educators need to adopt new and evolving pedagogies that incorporate AI to better support students at all levels of education. Finally, nursing students and practicing nurses must be equipped with the requisite knowledge and skills to effectively assess AIHTs and safely integrate those deemed appropriate to support person-centered compassionate nursing care in practice settings.

**International Registered Report Identifier (IRRID):**

RR2-10.2196/17490

## Introduction

### Artificial Intelligence

Artificial intelligence (AI) has been defined as technology that enables a computer system or computer-controlled robot to “learn, reason, perceive, infer, communicate, and make decisions similar to or better than humans” [1]. AI is interwoven in our everyday lives through our use of technologies such as cellular phones, smart televisions, and wearable fitness devices. New AI technologies are rapidly emerging, and within health systems, the use of AI health technologies (AIHTs) has become increasingly popular owing to their capacity for sorting and analyzing large amounts of research evidence, as well as clinical and patient data to identify patterns that enhance knowledge generation and decision making [2]. Based on these capabilities, AIHTs are predicted to transform various aspects of health systems in the coming decade.

In Canada, nurses represent the largest group of regulated health professionals, accounting for approximately 50% of the health workforce [3]. As AIHTs become more pervasive in the Canadian health system, it is predicted that nurses will function in greatly different roles and care delivery models [4]. These new roles and models will necessitate changes to nurses’ core competencies and educational requirements.

In the last 5 years, multiple expository papers and research studies have explored the current and predicted influences of AIHTs on nurse educators, nursing students, and practicing nurses [5-8]. Given the prediction that new technological advances are expected to transform aspects of nursing and its education [9,10], nurse educators need to increase their knowledge and comfort levels with both the concept and realities to be brought by emerging AIHTs. Additionally, nurses in clinical practice urgently require new knowledge and skills to effectively incorporate AIHTs into their practice [10].

### Background

As cited in the *Framework for the Practice of Registered Nurses in Canada*, “nursing knowledge is organized and communicated by using concepts, models, frameworks, and theories” [11]. There are four central concepts in particular that form the metaparadigm of nursing, and they are as follows: the person or client, the environment, health, and nursing [12]. Nurses use knowledge from a variety of sciences and humanities to inform their practice, including biology, chemistry, social and behavioral sciences, and psychology [11]. The integration of AIHTs into nursing education is essential to ensure nurses are adequately equipped with the requisite knowledge to optimize patient health outcomes in an evolving clinical and technological environment.

As emerging AIHTs modify health practices, health professionals will need to adapt their current ways of practicing to operationalize these technological advances [13]. Therefore, it is important for nurses to understand how AIHTs can be integrated into the conceptual foundation of nursing practice as they cocreate new models, frameworks, and theories that may be required to support the emerging technologies. This is particularly important given the increasing usage of AIHTs to enhance clinical decision making [14] and their potential to influence the traditional nurse-patient relationship.

Machine learning (ML), a subset of AI, uses algorithmic methodologies and techniques to process information in ways that can imitate human decision making [1]. Predictive analytics is a “branch of data analytics that uses various techniques, including ML, to analyze patterns in data and predict future outcomes” [15]. Clinical decision support systems that use AI-powered predictive analytics and ML algorithms to assist nurses in making clinical decisions for their patients based on trends in data are currently being used in clinical practice [16,17]. Similarly, virtual avatar apps that integrate chatbot technology to simulate interactive human conversations between health professionals and patients are growing in popularity [18,19]. Furthermore, social robots with natural language processing abilities [20] that enable them to understand, analyze, and manipulate data and generate language [14] are being increasingly used to provide additional companionship for residents in long-term care homes under the supervision of nurses. These technological advances are expected to cause considerable changes to the nursing landscape over the next decade [9], and nursing education as well as nurse educators will be at the forefront of these changes [21].

### Current State

Preparing nursing students and nurses for clinical practice in the age of AI requires a balance between teaching for current needs and anticipating future demands [9]. In the last two decades, there have been important accomplishments in nursing informatics that can be leveraged to provide curricular reform support for nurse educators [9]. For example, in 2004, the Technology Informatics Guiding Education Reform (TIGER) initiative was launched in the United States to provide resources to integrate technology and informatics into education, clinical practice, and research [22]. The *TIGER Nursing Informatics Competencies Model* was published in 2009 to support practicing nurses and nursing students [23]. Additionally, in 2012, the Canadian Association of Schools of Nursing (CASN) published the document, *Nursing Informatics Entry-to-Practice Competencies for Registered Nurses* [9,21,24]. Although these resources have been in existence for several years now, it is unclear if educators are effectively applying them and promoting their use [9,21]. A 2017 national survey of Canadian nurses found that the majority of respondents were unfamiliar with the CASN entry-to-practice informatics competencies [25]. According to Nagle et al [21] and Risling [9], one reason for the lack of uptake of these resources may be that a limited number of nurse educators possess the requisite knowledge, skills, and confidence themselves to address students’ learning associated with AI and digital health concepts. Transformation of nursing curricula will be necessary to ensure future nurses are equipped with informatics competencies, as well as competencies in digital and data literacy to work in clinical settings that increasingly use AI and ML technology. Strong nursing leadership will be required to incentivize nurse educators to embrace the need for curricular reform and to adopt new pedagogies that prepare nurses and nursing students to use these emerging technologies [10,26-30].

### Objectives

Considering the nascent topic of AIHTs and their influence on the nursing profession, it is important to understand the breadth and depth of literature that currently exists on this topic in order to prepare for future practice considerations. A scoping review was conducted to summarize the findings of four distinct research questions that explore the relationships between nurses, patients, and AIHTs [31]. A scoping review methodology was deemed appropriate for the aims of this project owing to its exploratory nature [32]. Given the number of articles included in the scoping review, a decision was made to divide the results into two standalone papers to improve clarity. This manuscript summarizes the findings of a research question that specifically addressed the current and predicted influences of AIHTs on nursing education. The results of the remaining three research questions have been published separately [33].

## Methods

### Scoping Review

This scoping review follows the methodological framework developed by Arksey and O’Malley [34] and further advanced by Levac et al [32], which delineates six steps to map the extent and range of material on a research topic [34]. The scoping review methodology helps to provide clarity on what is known and not known on a topic and situate this within policy and practice contexts [35]. The six steps included in the framework are as follows: (1) identifying the research question, (2) identifying relevant studies, (3) selecting the studies, (4) charting the data, (5) collating, summarizing, and reporting the results, and (6) consultation [34]. This scoping review was registered in the Open Science Framework database [36]. A scoping review protocol publication outlining the full methods of this review can be found elsewhere [31]. A steering committee, consisting of a person with lived experience and key stakeholders from various domains of nursing including nursing education, was convened to provide consultation throughout the project [31].

### Identifying the Research Questions and Relevant Studies

The research questions were co-developed by project team members and the steering committee. An information specialist was consulted in order to develop an effective search strategy [31]. This review details results from the following research question: what influences do emerging trends in AI-driven digital health technologies have, or are predicted to have, on nursing education across all domains? [31]. The databases of MEDLINE, Cumulative Index of Nursing and Allied Health Literature, Embase, PsycINFO, Cochrane Database of Systematic Reviews, Cochrane Central, Education Resources Information Centre, Scopus, Web of Science, and Proquest were searched for peer-reviewed literature using search strategies developed in consultation with the information specialist (Multimedia Appendix 1). A targeted website search was also conducted for pertinent grey literature, using Google search strings developed by the information specialist. Searches were limited to the last 5 years (ie, January 2014 to October 2019), after it was determined through consultation with the steering committee that the majority of literature on this emerging topic had been published within this time period [31].

### Study Selection

All peer-reviewed and grey literature results were downloaded into EndNote X7.8 (Clarivate Analytics) and imported into Distiller SR (Evidence Partners), a web-based systematic review software program used for screening. A screening guide was developed by two reviewers (CB and LH), and two levels of screening took place [31]. During title and abstract screening, articles were independently assessed by each reviewer and included if they were deemed relevant to the concepts of *AI* and *nursing* [31]. During second-level screening (full text relevance review), reviewers independently assessed each article to ascertain its relevance to one of the four research questions. The Joanna Briggs Institute suggests that when reporting inclusion criteria, they should be based on PCC elements (population, concept, and context) [37]. In terms of the population, articles that discussed nurses, nursing students, or nurse educators, or referred to health professionals more generally were included in this review if the information was relevant to nursing practice [31]. The core concept of this research question was AI and its influence on nursing education; therefore, in order to be included for this question, articles required a clear focus on AI and nursing education. The context and setting of focus included both clinical and academic settings. Finally, owing to the emerging nature of this topic, articles that only briefly discussed nursing education and AI were also included. Conflicts were resolved through discussion and consensus with a third party (RW) [31].

### Charting the Data

Standardized data charting forms were created by the two reviewers and tested with a representative sample of articles, with each reviewer independently charting the data [31]. Once consistency in data charting was achieved, data from each included full-text article were charted by one reviewer and verified by the second reviewer to ensure all relevant data were charted. Findings were recorded by study type in separate data charting forms for each research question (ie, qualitative versus quantitative study designs, and expository papers).

### Collating, Summarizing, and Reporting the Results

Once all the data from each included article were charted, the findings were summarized in the form of a data package and sent to members of the steering committee for review. Findings were organized by research question, with a table outlining overall descriptive findings of the included studies (ie, number of articles, setting, population, and types of AIHTs discussed). Additionally, categories were identified by the reviewers and outlined in a narrative fashion below the table of descriptive findings for each question.

### Consultation

The findings in the summary data package were discussed with the steering committee during two virtual meetings. Feedback was solicited to confirm the categories identified and their applicability or relevance to nursing education.

## Results

### Overview of Articles

**Figure 1:**
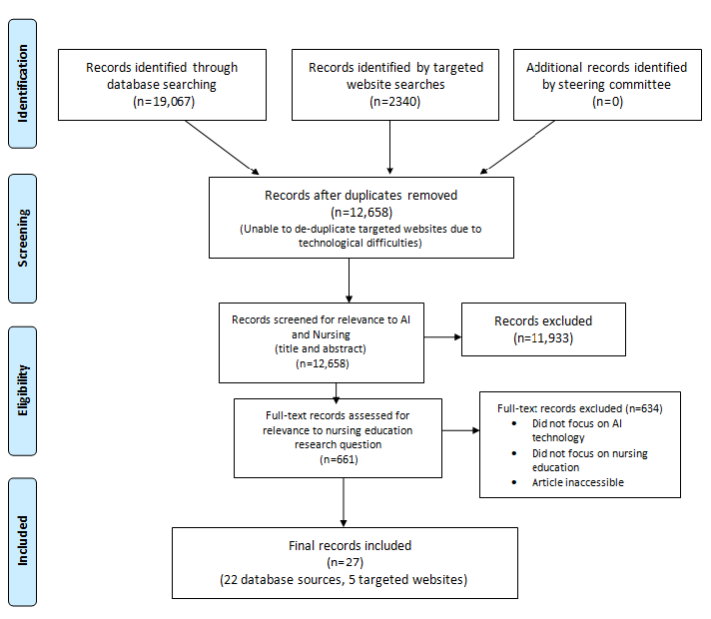
PRISMA flow diagram. AI: artificial intelligence.

A total of 27 articles were included for this research question; these were further characterized as 20 expository papers, six studies with quantitative or prototyping methods, and one qualitative study (see Figure 1 for the full Preferred Reporting Items for Systematic Reviews and Meta-Analyses [PRISMA] flow diagram [38]). The recipients of education included nursing students at the entry-to-practice, undergraduate, graduate, and doctoral levels, and practicing nurses in clinical settings. Faculty and instructors delivering educational content were referred to as nurse educators, nurse researchers, and nursing leaders. See Multimedia Appendix 2 for further details.

The types of emerging AIHTs discussed in the literature that have influenced or are predicted to influence nursing education included the following: virtual avatar apps (ie, chatbots) [7], smart homes [28], predictive analytics [27,39,40], virtual or augmented reality devices [41], and robots [26,42-45]. An overview of these emerging AIHTs and their current or predicted influences on nursing education is provided in Multimedia Appendix 2.

Specific examples of AIHTs that could be used as teaching tools in educational settings were also discussed. These included a face tracker system used to analyze nursing students’ emotions during clinical simulations [46] and ML wearable armbands used to measure the accuracy of students’ hand washing technique [47]. One article discussed a virtual patient gaming app used by nurse educators as an interactive teaching tool, providing students with virtual case scenarios congruent with the curriculum objectives [7]. One article encouraged the use of predictive analytics by nurse educators to enhance students’ clinical judgment and decision-making skills as they explore the executed decision path provided by the AIHT [40]. Finally, some articles simply presented a broad discussion of AIHTs and their potential influences on nursing education with no mention of specific examples [5,6,8-10,13,29,48-51].

The reviewers categorized the articles into the following two broad groups: (1) influences of AI on nursing education in academic institutions and (2) influences of AI on nursing education in clinical practice. The results of each of these categories and their subcategories are detailed in the ensuing paragraphs.

### Influences of AI on Nursing Education in Academic Institutions

#### Influences of AI on Nurse Educators

This scoping review revealed a growing trend in the use of AIHTs in nursing education in academic settings, which is expected to greatly increase in the near future. For instance, one article predicted that clinical simulation labs in these settings will have an increased presence of humanoid robots and *cyborgs* to complement their existing high-fidelity simulators [26]. Other emerging AIHTs in clinical simulation labs that were discussed in the literature included *face tracker* software, which uses ML to analyze students’ emotions during clinical simulations [46]. Authors noted that this type of technology allows nurse educators to assess the students’ emotions at each point of the simulation, along with the time spent on each component of the scenario [46]. The information gleaned through this process enables nurse educators to tailor the simulations to meet the students’ needs more effectively [46]. In addition, it was reported that this technology may help students to better understand emotion in their patients [46]. Finally, one article noted that in the foreseeable future, predictive analytics may be used to enhance students’ clinical judgment and decision-making skills as they analyze the executed decision path provided by the AIHT [40].

It is also predicted that virtual avatar apps, including virtual patient gaming apps and virtual tutor chatbots, may influence the delivery of nursing education in academic settings as educators use them as teaching tools to simulate interactive clinical scenarios and increase students’ comprehension of specific nursing concepts [7,50]. It was identified in the literature that these technologies have the potential to help students improve their communication skills with patients and the interprofessional team and enhance their confidence and self-efficacy prior to entering a real-life clinical environment [7]. Another AIHT that is expected to influence academic settings is a wearable armband that uses ML to evaluate a person’s hand washing technique [47]. Authors noted that nurse educators may use this technology to teach nursing students and practicing nurses in clinical settings proper hand washing techniques [47]. Finally, one author suggested that in the age of AI, ML could be used to analyze student data and create personalized learning pathways; this could assist nurse educators with student engagement and retention, and help meet their learning needs [50].

One article stated that the use of AIHTs to support learning in undergraduate nursing programs may positively influence nurses’ transition to practice by improving their clinical reasoning skills [41]. It is forecasted that students’ exposure to AIHTs in their undergraduate clinical experiences may help prepare them for jobs in technology-rich clinical settings [45]. For example, AIHTs that incorporate virtual or augmented reality provide students with an innovative approach to experiencing the clinical environment [41]. Another article suggested that nursing students are responsive and receptive to virtual reality education modalities and virtual reality training may be more effective than traditional teaching modalities in some situations [41]. Given these potential benefits, several authors urge nurse educators to consider the value of adopting new pedagogies that provide opportunities for undergraduate nursing students to engage with these emerging technologies [6,10,26-29].

There was minimal literature discussing the influence of AIHTs on the delivery of nursing education at the postgraduate level specifically. One article noted that nursing faculty (ie, at the postgraduate level) will need to know how to use specialized data science methods, and understand how to identify policy trends and implications related to these methods to bring value to nursing science [5]. The authors noted that big data should be used by educators to make nursing knowledge more accessible, visible, visually interesting, and data enhanced [5], both in the classroom and beyond.

The emergence of AIHTs in nursing is predicted to shift nurse educators toward a more multidisciplinary teaching approach (ie, nurses working collaboratively with information technologists, robotics experts, and computer programmers) [26]. One article noted that these types of collaborations have the potential to bridge the skills gaps in nursing and support the advancement of professional groups such as clinical data scientists, medical software engineers, and digital medicine specialists [48], and nurses could then explore these roles.

#### Influences of AI on Nursing Students

Several articles have highlighted the need for a focused transformation of undergraduate nursing curricula to ensure future nurses are equipped to work in clinical settings that increasingly use AIHTs [6,10,26-29]. Risling [9,49] purports that informatics should be a required nursing competency and that nursing curricula should include core courses on this topic. Others have suggested that nursing curricula should be redesigned to include topics such as data literacy, technological literacy, systems thinking, critical thinking, genomics and AI algorithms, ethical implications of AI, and analysis and implications of big data sets [6,48,52].

Curricular revisions are also delineated in the literature for graduate-level nursing courses to integrate more advanced AI content on topics such as informatics, ethics, privacy, research, and engineering concepts [5,28,39,48,49]. In one article, authors noted that smart homes are expected to influence graduate nursing curricula as they grow in popularity [28]. It is predicted that students will need to understand how AI smart home technology uses sensor data to assist older adults with “aging in place” by monitoring their movement in the home [28].

Changes are also suggested for courses at the doctoral level to provide more in-depth opportunities for nurses to develop competencies in predictive modeling, biostatistical programming, data management, risk adjustment, multivariable regression, ML, governance of big data, and cyberthreats [5,39]. Two universities in the United States have strategically incorporated data science into the core curriculum for their nursing doctoral program [5]. It was noted that the integration of data sciences with nursing theory development will be an important addition to the curriculum at the postgraduate level in these universities as more AIHTs are being used in the health system [5].

In addition to the need for new AI technological competencies, several authors accentuated the importance of a continued focus on interpersonal human communication skills and empathy in nursing education curricula. This combined focus is deemed necessary to ensure that nurses continue to provide person-centered compassionate care in a health system increasingly being dominated by machines [13,27].

Innovative educational programs that combine biomedical engineering and nursing have been proposed as a way to educate a new cadre of health professionals and increase opportunities for nurses to contribute to the co-design of AIHTs [53]. At the time this scoping review was conducted, no universities had created an entirely new discipline to support the anticipated nursing-AI integration (eg, *nurse-engineering*); however, a few universities had created unique collaborations or joint degrees to improve patient experiences or health system efficiencies with greater use of technology [53].

### Influences of AI on Nursing Education in Clinical Practice

The majority of articles in this category focused on the influences of AI on nurses within the hospital setting. However, some publications focused on the influences of AI on nurses in long-term care or home care settings as well. Given the scope of change that AIHTs are likely to engender, authors have recommended that nurse educators in all practice settings provide appropriate professional development education to equip nurses with the requisite knowledge and skills to use these tools in their work environment [8]. It has also been suggested that nurses assume responsibility for upgrading their skills as AIHTs are increasingly deployed in clinical practice settings [10,29,42-44].

It was predicted in the literature that more professional development opportunities (eg, courses and workshops) will be needed in the workplace to support emerging areas of AIHTs [8,29,54] to ensure nurses maintain relevant competencies and skills in their practice setting [10]. One article suggested that nursing informaticians should be utilized to establish a strong foundation of evidence regarding the necessity of nursing data [8], which can be used to inform professional development workshops and nursing clinical competencies. Furthermore, two articles suggested that educational resources be tailored to recipients [48,52]. For example, educational resources to “educate the educators” [48] will differ from those used to train point-of-care nurses in their clinical settings [52], and continued professional development will need to be tailored to those specialists who work more intimately with AIHTs (eg, nursing informaticians) [48,52]. One article suggested that in the clinical setting, examining predictive analytics models can help facilitate knowledge transfer and build capacity in newer less experienced nurses to understand AI’s personalized decision-making process [40].

## Discussion

### Key Considerations

AIHTs are already beginning to influence the nursing practice, and it is crucial that nurse educators are prepared to equip nurses and nursing students to integrate AIHTs effectively into practice. Considerable curricular reform is needed at all education levels and all designations to support this paradigm shift, and this includes entry-to-practice, undergraduate, graduate, and doctoral education. This reform must ensure that nurses and nursing students are educated on emerging topics that are relevant to AI, based on their roles and responsibilities. Recommended topics of education included the following: basic informatics competencies [8,9,26,28,44], data analytics, predictive modeling and ML principles [5,10,27,39,51,52], engineering principles [26,42,52,53] digital/data literacy [6,48], ethics [5,9,28,48,51,52], privacy issues (including security breaches or “cyberthreats”) [5,9], big data governance [5,48,52], technocentric cultural competence [26], AI research design [28], and robotics care and operations [26,42].

Efforts to align nursing education with this paradigm shift should also include new pedagogies that support emerging AIHTs [6]. Incorporating these technologies into nursing education can increase familiarity and comfort for students when they enter the clinical practice setting [6]. As suggested by Murray [6], the nursing profession is entering an inflection point where AIHTs may enhance various aspects of nursing practice and catalyze much needed changes in contemporary nursing education. Nurse educators, practicing nurses, and students need to remain actively engaged in the planning and implementation of these technologies, thereby enhancing opportunities for their successful integration.

### Future State: Nursing Leadership Requirements

Nurse educators in both clinical practice settings and academic institutions have an essential leadership role in preparing nurses and nursing students for a future that will certainly include a wide variety of AIHTs. In order to support a technologically proficient nursing workforce, educators must create a learning environment conducive to nurses evolving their understandings of the novel relationships that exist among nurses, patients, and AIHTs [55]. An important first step will be embedding informatics and digital health technology competencies into all areas of nursing education. A solid understanding of these principles will ensure nurses are equipped to use AIHTs in their clinical practice and, perhaps even more importantly, have the potential to be valuable contributors to the ongoing development of these technologies (ie, co-designers). It has been suggested that the AI industry would benefit from hiring experts from various health disciplines to engage in design processes, and the nursing profession has the potential to provide this expertise [39].

In order to facilitate such a substantial shift, curricula will need to be assessed for their contemporary relevance to health care realities and for their ability to proactively prepare nursing for the future demands of AIHTs [30,56]. One way of accommodating this will be to develop curricula that address the need for a new specialty, the *nurse-engineer* role, to develop a nurse’s role as a co-designer of AIHTs. Undergraduate nursing programs that combine nursing principles with engineering principles can advance the development of AIHTs and help nurses understand the principles behind the AIHTs that they will likely encounter in clinical settings [26,42,53]. The involvement of nurses in co-design of these AIHTs at all stages of design, implementation, and evaluation will reduce the risk of creating technology that burdens health professionals and will help to prevent costly mistakes that arise from lack of clinician input [29,45]. Once again, in order for this to happen, nursing leadership will be required to equip nurses with knowledge and skills in informatics, digital literacy, engineering, and ML in their preliminary nursing education.

Nurses, especially those involved in co-design, must also be prepared to address the nuanced privacy, equity, and ethical implications that will likely arise from the use of AIHTs in nursing practice. Nursing curricula should discuss ethical concerns such as data breaches, the potential for bias in the data used to develop algorithms, and the importance of social justice and person-centered approaches in the design of AIHTs [5,9,30].

In addition to the proposed curricular revisions discussed above, authors also stressed the importance of placing continued emphasis on therapeutic relationships and interpersonal communication in nursing education, as these are core values of nursing care that differentiate nursing from AIHTs [13]. A continued focus on these core nursing values will serve to equip students with the skills necessary to convey compassion and empathy in technology-rich health systems. Nurses and nursing students must begin to reflect on the ways AIHTs may impact nurse-patient interactions and communication patterns between patients, caregivers, and other members of the interprofessional team [13]. Fernandes et al [13] stated, “the transformation of curricula and professional practice focusing on interpersonal and intrapersonal intelligence with attitudes that value human skills will ensure nursing’s place/role in a society dominated by machines and scientific progress.”

### Empowering Nurses and Nursing Students

It has been forecasted that in the immediate future, nurses may use predictive analytics to prioritize educational topics for their patients before discharge [57]. It is also likely that nurses will use virtual avatar apps with chatbot technology to assist in providing patients with additional education, coping strategies, and mental health supports [58]. Building deeper awareness and sensitivity around the implications of these AIHTs through nursing education is a pragmatic first step toward the eventual goal of developing competency and expertise across all domains of nursing practice, and in all settings. This education should be provided in both academic settings (for nursing students) and in clinical practice settings (for practicing nurses) through professional development opportunities such as courses and workshops [8].

Along with building deeper awareness of the topic, nursing students must be empowered to re-envision health practices of the future, as it is clear that these forms of advanced technology will likely change traditional nursing processes and ways of knowing. Furthermore, the emergence of AIHTs demands changes in the usual way of conducting nursing education. Emerging technologies have accentuated the need for nurse educators to reflect on past practices and transition toward new ways of engaging students [6]. However, in order for new models of nursing education to be successful, both educators and students must be receptive to sizable changes likely to occur with the scaling of AIHTs in all areas of health systems. Subsequently, for nursing education to evolve successfully, both students and educators must appreciate the transformative nature of AIHTs, and their direct and indirect impacts upon all aspects of health delivery and nursing education [26].

While the receptivity of nursing education toward appreciating the growing ubiquity of AIHTs varies among health professionals and educators, ensuring the various fundamental tenets of nursing are not minimized or diluted will be essential moving into the future. For instance, the role of compassionate care within nursing practice should be viewed as an important and requisite feature of all care provided through or with AIHTs that are used by nurses. The nursing profession must not lose sight of its greatest attributes, including compassionate care, in light of a technological future [13]. Concerns related to nurse-patient interactions and therapeutic relationships will be paramount in the years to come, and nurses require the skills to balance human caring needs with technological AI advancements [9]. While technology and nursing are inextricably linked in nursing practice, the caring values espoused by nurses must be protected and amplified through the technology used to support care delivery [44].

### Future Research

While discussions about AI are beginning to emerge in the nursing education literature, many of the articles included in this review focused on nursing informatics more generally and briefly mentioned AI. Additionally, as the majority of papers included in this review were expository papers and white papers, there is a need for more research in this context. Further research is needed to continue identifying the educational requirements and core competencies necessary for specifically integrating AIHTs into nursing practice. Future research should also focus on identifying the most effective ways AI can be used as a tool in nursing education.

### Limitations

The findings of this review should be interpreted in light of some limitations. Computer science and engineering databases were not searched owing to accessibility issues and organizational licensing restrictions. This limitation may have led to research gaps, and it is recommended that future reviews on the topic of AI and nursing utilize these databases. In addition, only articles published in English were considered for selection and the reference lists of included studies were not searched. This may have led to important articles on the topic being missed. The reviewers did not use Cohen kappa when calculating interrater agreement during title and abstract screening, and instead used percentage agreement (97% agreement). While this was done for feasibility purposes, it is recognized that percentage agreement is not as reliable as Cohen kappa when calculating interrater agreement. Finally, the authors acknowledge the likelihood that more research has been conducted on this topic since performing the original search in 2019; however, owing to feasibility restrictions, it was not possible to perform an updated search.

### Conclusions

Nurse educators in clinical practice and academic institutions around the world have an essential leadership role in preparing nurses and nursing students for the future state of AIHTs. It is evident that AIHTs are transforming heath systems as they currently exist, and the nursing profession needs to be actively involved in this rapidly evolving process or risk unwanted consequences for both patients and the discipline if this technological revolution proceeds unchecked. Nurse educators need to prepare the profession for a future that in many institutions and settings is already here.

AIHTs are destined to transform health education and delivery, and this process will require education, preparation, and adoption by nurse educators, as well as a strong amount of co-design of these technologies. In collaboration with other health disciplines, nurses are in an ideal position to lead research on AIHTs. Nurses uniquely understand the complexities of the health environment [45] and can identify the ways patients are best served by technology [49]. A strong educational foundation in AI principles is the first step to ensuring nurses’ contribution at all levels of design, implementation, and evaluation of AIHTs.

To our knowledge, this is the first scoping review to examine AIHTs and their influence on nursing education. While there has been research conducted on AIHTs and on nursing education as separate research topics, now is the time to realize the critical relationship between these two entities. AIHTs cannot be implemented in an effective manner without the solid foundation of nursing education, in both academic and clinical practice settings. The findings of this review will help nurse educators across all sectors to proactively shape the nursing-AI interface, ensuring that nursing education aligns with core nursing values that promote compassionate care.

## Appendix

Multimedia Appendix 1 MEDLINE and targeted website search strategies.

Multimedia Appendix 2 Overview of the findings.

## Abbreviations

AI: artificial intelligence
AIHT: artificial intelligence health technology
CASN: Canadian Association of Schools of Nursing
ML: machine learning
TIGER: Technology Informatics Guiding Education Reform
