# Supplementary material for: Predicted Influences of Artificial Intelligence on Nursing Education: Scoping Review
Source: JMIR Nurs. 2021 Jan 28;4(1):e23933. doi: 10.2196/23933 (PMC8328269; doi:10.2196/23933)
Supplement: Multimedia Appendix 1 [file nursing_v4i1e23933_app1.docx]

**Targeted Website Search**

The following 5 search strings were developed in consultation with the information specialist and were used for all targeted website searches:

1. (“artificial intelligence" | robot | robots | robotics | digital technologies) AND (nurse | nurses | nursing) AND site:___
2. ("artificial intelligence" | robot | robots | robotics) AND (nurse | nurses | nursing) AND site:___
3. (“artificial intelligence” | robot | robots | robotics | digital technologies) AND (nurse | nurses | nursing) AND (compassion | compassionate | humanistic | empathy | therapeutic | patient-centered) AND site: ____
4. (“artificial intelligence” | robot | robots | robotics | digital technologies) AND (nurse | nurses | nursing) AND ("co-design" | codesign | design | participate | develop) AND site: ____
5. (“artificial intelligence” | robot | robots | robotics | digital technologies) AND (nurse | nurses | nursing) AND (educate |education | school | curriculum) AND site: ____

Websites used:

1. World Health Organization (WHO) (site:.who.int)

2. National Health Service (NHS) (site:.nhs.uk)

3. Office of the National Coordinator for Health Information Technology (ONC) (site:.health.it.gov)

4. Institute for Research on Healthy Public Policy (IRPP) (site:.irpp.org)

5. Canada Health Infoway (site:.infoway-inforoute.ca)

6. Canadian Association of Schools of Nursing (CASN) (site:.casn.ca)

7. Healthcare Information and Management Systems Society (HIMSS) (site:.himss.org)
