# Supplementary material for: Predicted Influences of Artificial Intelligence on Nursing Education: Scoping Review
Source: JMIR Nurs. 2021 Jan 28;4(1):e23933. doi: 10.2196/23933 (PMC8328269; doi:10.2196/23933)
Supplement: Multimedia Appendix 2 [file nursing_v4i1e23933_app2.docx]

Multimedia Appendix 2: Overview of Findings

| **Research Question** | **Types of Articles (n=27)** | **Country of Publication** | **Population** | **Current or Predicted influences of AIHTs on Nursing Education Identified in the Literature** |
| --- | --- | --- | --- | --- |
| What influences do emerging trends in AI-driven digital health technologies have, or are predicted to have, in nursing education across all domains? | 20 expository papers/white papers [1, 2-20]  6 studies with quantitative or prototyping methods [21-26]  1 qualitative study [27] | Brazil [6, 23], USA [1, 2, 8, 13-15, 17-19, 22, 26], Germany [20, 24],  Korea [25], Canada [3, 4, 12], Sweden [16], Japan [7], Philippines [5], Singapore [21], Taiwan [27], UK [9-11] | **Recipients of education:**  Nursing students at the entry-to-practice or undergraduate level [2, 5, 7, 8, 12, 13, 19, 21-23]  Graduate and doctoral level nursing students [1, 12, 14, 17 22)  Nursing students (level of education not specified) [3, 4, 6, 15, 18, 24]  Nurses in clinical practice [5, 6, 16, 24-27]  Health professionals (generally) [9-11, 20]  **Instructors:**  -Some articles discussed ‘nursing researchers’ and ‘nursing leaders’ [3, 18]  -nurse educators [2, 4, 7, 15, 19, 22] | ***Virtual Patient Gaming App [21]:***   - One article stated that undergraduate nursing students had access to a virtual patient gaming app that used chatbot technology [21] - Four case scenarios were developed within the app congruent with the nursing students’ learning objectives [21] |
|  |  |  |  | ***Smart Homes [22]:***   - Authors noted that smart homes are expected to influence graduate nursing curricula as they grow in popularity [22]. - It is predicted that students will need to understand how AI uses sensor data and smart home technology to assist older adults with aging-in-place by monitoring their movement in the home [22]. |
|  |  |  |  | ***Predictive Analytics [8, 14, 25]:***   - The literature noted that in the foreseeable future predictive analytics may be used to enhance students’ clinical judgment and decision-making skills as they analyze the executed decision path provided by the AIHT [25] |
|  |  |  |  | ***Robots [7, 15, 16, 27, 18]:***   - Authors stated that nurses will require knowledge of basic engineering concepts, enabling them to assess the safety of robotic devices, help clients and their families select appropriate technology for their home, and troubleshoot common technology malfunctions [15] - Individual faculty course delivery may transition to collaborative learning approaches that support interactions between nursing students and students from other disciplines such as information and communication technology, robotics, and computer programming to increase students’ knowledge and technology competence [7] - Robotics may be used in clinical simulation labs as a teaching tool [7] |
|  |  |  |  | ***Face Tracker System [23]:***   - In one study educators used this AIHT to analyze nursing students’ emotions during a clinical learning simulation activity [23] - This AIHT may influence clinical simulation labs as educators use it to determine which areas of the simulation students felt confident in, and which areas caused them difficulty [23] |
|  |  |  |  | ***ML Wearable Armbands [24]:***   - This AIHT was used to evaluate students’ hand washing technique [24] - Its utility as a teaching tool for nurse educators in both academic institutions and clinical practice settings is expected to increase. |
|  |  |  |  | ***AIHTs that Incorporate Virtual Reality [26]:***   - AIHTs that incorporate virtual or augmented reality are expected to influence nursing curricula by providing educators with innovative approaches to influence cognitive development - This article suggests that nursing students are responsive and receptive to virtual reality education modalities and virtual reality training may be more effective than traditional teaching modalities in some situations [26] |
|  |  |  |  | ***Broad Discussion of AIHTs [1-6, 9, 10, 12, 19, 20]:***   - Authors discussed potential uses for AIHTs in nursing education and suggested curriculum changes (ie, content on ethics, privacy [1, 3], cyberthreats [1], data literacy [2] |

References:

1. Gephart SM, Davis M, Shea K. Perspectives on Policy and the Value of Nursing Science in a Big Data Era. Nursing Science Quarterly. 2018;31(1):78-81. PMID: 29235962
2. Murray TA. Nursing Education: Our Iceberg Is Melting. Journal of Nursing Education. 2018;57(10):575-6. PMID: 30277540
3. Risling TL, Low C. Advocating for Safe, Quality and Just Care: What Nursing Leaders Need to Know about Artificial Intelligence in Healthcare Delivery. Nursing leadership (Toronto, Ont ). 2019;32(2):31-45.
4. Risling T. Educating the nurses of 2025: Technology trends of the next decade. Nurse Education in Practice. 2017;22:89-92. PMID: 28049072
5. Pepito JA, Locsin R. Can nurses remain relevant in a technologically advanced future? International Journal of Nursing Sciences. 2019;6(1):106-10. PMID: 31406875
6. Fernandes MNF, Esteves RB, Teixeira CAB, Gherardi-Donato E. The present and the future of Nursing in the Brave New World. Revista Da Escola de Enfermagem Da Usp. 2018;52:e03356. PMID: 30043931
7. Tanioka T, Yasuhara Y, Dino MJS, Kai Y, Locsin RC, Schoenhofer SO. Disruptive Engagements With Technologies, Robotics, and Caring: Advancing the Transactive Relationship Theory of Nursing. Nursing Administration Quarterly. 2019;43(4):313-21. doi: 10.1097/NAQ.0000000000000365
8. Lynn LA. Artificial intelligence systems for complex decision-making in acute care medicine: a review. Patient Safety in Surgery. 2019;13:6. DOI: <https://doi.org/10.1186/s13037-019-0188-2>
9. Foley T, Woollard J. The digital future of mental healthcare and its workforce: A report on mental health stakeholder engagement to inform the Topol Review. Health Education England; 2019. Retrieved from <https://topol.hee.nhs.uk/the-topol-review/>
10. NHS Health Education England. The Topol review interim report: Preparing the health care workforce to deliver the digital future. 2018. Retrieved from <http://allcatsrgrey.org.uk/wp/download/education/medical_education/Topol-Review-interim-report_0.pdf>
11. Secretary of State for Health and Social Care. The Topol review: Preparing the health care workforce to deliver the digital future. NHS; 2019. Retrieved from <https://topol.hee.nhs.uk/the-topol-review/>
12. Risling T. Why AI needs nursing: Policy Options; 2018. Retrieved from <https://policyoptions.irpp.org/magazines/february-2018/why-ai-needs-nursing/>.
13. Glasgow MES, Colbert A, Viator J, Cavanagh S. The Nurse-Engineer: A New Role to Improve Nurse Technology Interface and Patient Care Device Innovations. Journal of Nursing Scholarship. 2018;50(6):601-11. PMID: 30221824
14. Linnen DT, Javed PS, D'Alfonso JN. Ripe for Disruption? Adopting Nurse-Led Data Science and Artificial Intelligence to Predict and Reduce Hospital-Acquired Outcomes in the Learning Health System. Nursing Administration Quarterly. 2019;43(3):246-55. PMID: 31162343
15. Sharts-Hopko NC. The coming revolution in personal care robotics: what does it mean for nurses? Nursing Administration Quarterly. 2014;38(1):5-12. PMID: 24317027
16. Salzmann-Erikson M, Eriksson H. Letter to the Editor: Prosperity of nursing care robots: an imperative for the development of new infrastructure and competence for health professions in geriatric care. Journal of Nursing Management. 2017;25(6):486-8. PMID: 28544354
17. Henly SJ, McCarthy DO, Wyman JF, Heitkemper MM, Redeker NS, Titler MG, et al. Emerging areas of science: Recommendations for Nursing Science Education from the Council for the Advancement of Nursing Science Idea Festival. Nursing Outlook. 2015;63(4):398-407. PMID: 26187079
18. Clipper B, Batcheller J, Thomaz AL, Rozga A. Artificial Intelligence and Robotics A Nurse Leader's Primer. Nurse Leader. 2018;16(6):379-84. DOI: <https://doi.org/10.1016/j.mnl.2018.07.015>
19. Skiba DJ. Horizon report: Knowledge obsolescence, artificial intelligence, and rethinking the educator role. Nursing Education Perspectives. 2017;38(3):165-7. DOI: 10.1097/01.NEP.0000000000000154
20. European Observatory on Health Systems and Policies & Beck, Jan-Philipp. (‎2019)‎. Are we ready for AI? Why innovation in tech needs to be matched by investment in people. Eurohealth, 25 (‎3)‎, 9 - 11. World Health Organization. Regional Office for Europe. Retrieved from <https://apps.who.int/iris/handle/10665/332507>
21. Shorey S, Ang E, Yap J, Ng ED, Lau ST, Chui CK. A virtual counseling application using artificial intelligence for communication skills training in nursing education: Development study. Journal of Medical Internet Research. 2019;21(10). PMID: 31663857
22. Fritz RL, Dermody G. A nurse-driven method for developing artificial intelligence in "smart" homes for aging-in-place. Nursing Outlook. 2019;67(2):140-53. PMID: 30551883
23. Mano LY, Mazzo A, Neto JRT, Meska MHG, Giancristofaro GT, Ueyama J, et al. Using emotion recognition to assess simulation-based learning. Nurse Education in Practice. 2019;36:13-9. DOI: 10.1016/j.nepr.2019.02.017
24. Kutafina E, Laukamp D, Jonas SM. Wearable Sensors in Medical Education: Supporting Hand Hygiene Training with a Forearm EMG. Studies in Health Technology & Informatics. 2015;211:286-91. PMID: 25980884
25. Afzal M, Hussain M, Ali Khan W, Ali T, Lee S, Huh EN, et al. Comprehensible knowledge model creation for cancer treatment decision making. Computers in Biology & Medicine. 2017;82:119-29. PMID: 28187294
26. Sitterding MC, Raab DL, Saupe JL, Israel KJ. Using Artificial Intelligence and Gaming to Improve New Nurse Transition. Nurse Leader. 2019;17(2):125-30. DOI: <https://doi.org/10.1016/j.mnl.2018.12.013>
27. Liang HF, Wu KM, Weng CH, Hsieh HW. Nurses' Views on the Potential Use of Robots in the Pediatric Unit. Journal of Pediatric Nursing. 2019;47:e58-e64. PMID: 31076190
